# Efficacy of Intravenous, Endotracheal, or Nasal Adrenaline Administration During Resuscitation of Near-Term Asphyxiated Lambs

**DOI:** 10.3389/fped.2020.00262

**Published:** 2020-06-02

**Authors:** Nils T. Songstad, Claus Klingenberg, Erin V. McGillick, Graeme R. Polglase, Valerie Zahra, Georg M. Schmölzer, Peter G. Davis, Stuart B. Hooper, Kelly J. Crossley

**Affiliations:** ^1^Department of Paediatric and Adolescent Medicine, University Hospital of North Norway, Tromsø, Norway; ^2^Paediatric Research Group, UiT-The Arctic University of Norway, Tromsø, Norway; ^3^The Ritchie Centre, Hudson Institute of Medical Research Monash University, Melbourne, VIC, Australia; ^4^Department of Obstetrics and Gynaecology, Monash University, Melbourne, VIC, Australia; ^5^Centre for the Studies of Asphyxia and Resuscitation, Royal Alexandra Hospital, Edmonton, AB, Canada; ^6^Department of Pediatrics, University of Alberta, Edmonton, AB, Canada; ^7^Neonatal Services and Newborn Research Centre, The Royal Women's Hospital, Melbourne, VIC, Australia; ^8^Department of Obstetrics and Gynecology, The University of Melbourne, Melbourne, VIC, Australia; ^9^Murdoch Childrens Research Institute, Melbourne, VIC, Australia

**Keywords:** adrenaline, resuscitation, newborn lamb, perinatal asphyxia, administration route

## Abstract

**Objectives:** Neonatal resuscitation guidelines recommend administering intravenous (IV) adrenaline if bradycardia persists despite adequate ventilation and chest compressions (CC). Rapid IV access is challenging, but little evidence exists for other routes of administration. We compared IV, endotracheal (ET), and intranasal routes for adrenaline administration during resuscitation of asphyxiated newborn lambs.

**Study design:** Near-term lambs (*n* = 22) were delivered by caesarean section. Severe asphyxia was induced by clamping the umbilical cord while delaying ET ventilation until blood flow in the carotid artery ceased. Following a 30 s sustained inflation and ventilation for 30 s, we commenced uncoordinated CC at 90/min. We randomized four groups receiving repeated treatment doses (Tds) every 3rd min of (i) IV-Adrenaline (50 μg), (ii) ET-Adrenaline (500 μg), (iii) Nasal-Adrenaline via an atomizer (500 μg), and (iv) IV-saline. If return of spontaneous circulation (ROSC) was not achieved after three Tds by the assigned route, up to two rescue doses (Rds) of IV adrenaline were administered. Main outcome measures were achievement of ROSC and time from start of CC to ROSC, defined as heart rate >100/min, and mean carotid arterial pressure >30 mmHg.

**Results:** In the IV-Adrenaline group, 5/6 lambs achieved ROSC after the first Td, whereas 1 lamb required two Tds before achieving ROSC. In the ET-Adrenaline group, 1/5 lambs required one Td, 1 lamb required three Tds, 2 lambs required 2 Rds, and 1 did not achieve ROSC. In the Nasal-Adrenaline group, 1/6 lambs required one Td, 2 required two Tds, whereas 3 lambs required either one (2 lambs) or two (1 lamb) Rds of adrenaline to achieve ROSC. In the IV-saline group, no lambs achieved ROSC until adrenaline Rds; 4/5 lambs required one Rd and 1 lamb required two Rds. Time to ROSC was shorter using IV-Adrenaline (2.4 ± 0.4 min) compared with ET-Adrenaline (10.3 ± 2.4 min), Nasal-Adrenaline (9.2 ± 2.2 min), and IV-saline (11.2 ± 1.2 min).

**Conclusion:** IV adrenaline had superior efficacy compared with nasal or ET administration. Nasal administration had a similar effect as ET administration and is an easier route for early application. Nasal high-dose adrenaline administration for neonatal resuscitation merits further investigation.

## Introduction

Approximately 3–6% of all newborns require tactile stimulation and assisted ventilation at birth in order to aerate their lungs and commence pulmonary gas exchange (1–3). These measures form the cornerstone of neonatal resuscitation, and most infants require no further interventions. Less than 0.1% of term and near-term infants receive advanced resuscitation in the form of chest compressions (CC) with or without adrenaline administration (3, 4). Advanced resuscitation is associated with a poor prognosis, which raises questions as to whether improved protocols can lead to better outcomes (4).

The cerebral blood vessels of acidotic, severely asphyxiated infants are maximally dilated in an attempt to sustain cerebral oxygen delivery, but myocardial function is severely depressed (5). In asystolic or severely bradycardic newborns, the return of spontaneous circulation (ROSC) usually follows an increase in diastolic pressure, which is thought to re-establish coronary perfusion pressure (4, 6). Current neonatal resuscitation guidelines recommend that if the heart rate remains below 60 beats per min (bpm) and CCs are ineffective, then adrenaline should be administered. The preferred route of adrenaline administration is intravenous (IV), via an umbilical venous catheter (1, 7). Adrenaline is thought to increase peripheral vasoconstriction, elevate diastolic pressure, and thereby increase coronary perfusion pressure. In animal models of neonatal asphyxia, effective CCs with compression rates of 90/min usually do not generate sufficiently high diastolic pressures to achieve ROSC (8, 9). The addition of adrenaline is therefore required to enable successful resuscitation (8).

As adrenaline is used infrequently and advanced resuscitation is very challenging in a clinical research setting, rigorous human neonatal studies investigating adrenaline administration and dosing are extremely difficult. Thus, the evidence base for appropriate dosing, order, and route of administration remains controversial and relies on data extrapolated largely from adult or post-transitional animal studies (10, 11). Establishing an IV route often takes several minutes, even with a trained resuscitation team (12). Therefore, both the European and the US resuscitation guidelines suggest that endotracheal (ET) administration of adrenaline may be used in higher dosages (50–100 μg/kg) during neonatal resuscitation (7, 13). However, both guidelines note the lack of supportive data. Moreover, emergency ET intubation is difficult and even with a trained resuscitation team it may also take minutes before the ET tube is in place (12). A simpler route could lead to earlier adrenaline administration in cases of severe asphyxia not responding to ventilation and CCs.

The aim of this study was to compare the effects of IV and ET adrenaline administration with a novel approach of administering adrenaline nasally via an atomizer spray during resuscitation of near-term asphyxiated lambs.

## Materials and Methods

The study protocol was approved by the Monash University, Animal Ethics Committee (MMCA). All experiments were performed according to animal ethical guidelines, in compliance with the ARRIVE guidelines (14) and the Australian National Health and Medical Research Council code of practice for the care and use of animals for scientific purposes.

### Animal Preparation

We used an established lamb model of near-term asphyxia (15). Pregnant ewes at 139 ± 2 days of gestation (term ~147 days) were anesthetized, and the fetal head and neck were exposed via caesarean section. Polyvinyl catheters were inserted into the left carotid artery for blood pressure (BP) measurement, the left jugular vein for IV infusions, and a femoral artery for blood sampling. Ultrasonic flow probes (3PS; Transonic Systems, Ithaca, NY) were placed around the right carotid artery and the left pulmonary artery, as previously described (16). Preductal fetal SpO_2_ was measured at the right forelimb using a transcutaneous pulse oximeter (Masimo, Irvine, CA), and FiO_2_ used for ventilation of the ewe was titrated to achieve a fetal SpO_2_ of 50–55%. The fetal trachea was orally intubated with a cuffed ET tube (4.0 or 4.5 mm) which was clamped to avoid breathing or gasping.

### Asphyxiation and Initial Resuscitation Procedures

Lung liquid was drained passively before the umbilical cord was clamped and cut. The lambs were delivered, dried, and placed on a resuscitation table under a radiant heater to maintain normal body temperature (CosyCot, Fisher and Paykel, Auckland, New Zealand). Asphyxia was induced by delaying ET ventilation until blood flow in the carotid artery ceased. The ET tube clamp was then removed, and lung aeration commenced with a 30 s sustained inflation at 35 cmH_2_O delivered with a T-piece device (Neopuff, Fisher & Paykel, Auckland, New Zealand) using air. Lambs were then ventilated for 30 s using a peak inflation pressure of 35 cmH_2_O, positive end expiratory pressure of 5 cmH_2_O, inflation time of 0.5 s at rate of 60/min, and FiO_2_ 1.0 (Dräger Babylog 8000 plus, Lübeck, Germany) before uncoordinated CC at a rate of 90/min commenced.

### Treatments

Prior to delivery, 22 lambs were randomly assigned to one of four treatment groups:

1. 0.5 mL (50 μg) of adrenaline (0.1 mg/mL, Linkpharma, UK), given IV followed by 5.0 mL of saline flush; IV-Adr (*n* = 6).
2. 5 mL (500 μg) of adrenaline (0.1 mg/mL, Linkpharma, UK) into the trachea, via a nasogastric feeding tube down the ET tube; ET-Adr (*n* = 5).
3. 0.25 mL (250 μg) of adrenaline (1 mg/mL, Aspen Pharmacare, NSW, Australia) applied into each nostril (500 μg total dose) using an Intranasal Mucosal Atomization Device (LMA MAD Nasal™, Teleflex, Morrisville, USA). Nasal administration was preceded by suctioning of both nostrils; Nasal-Adr (*n* = 6).
4. IV saline control group receiving 5.0 mL of saline via the jugular vein; IV-saline (*n* = 5).

**Figure 1:**
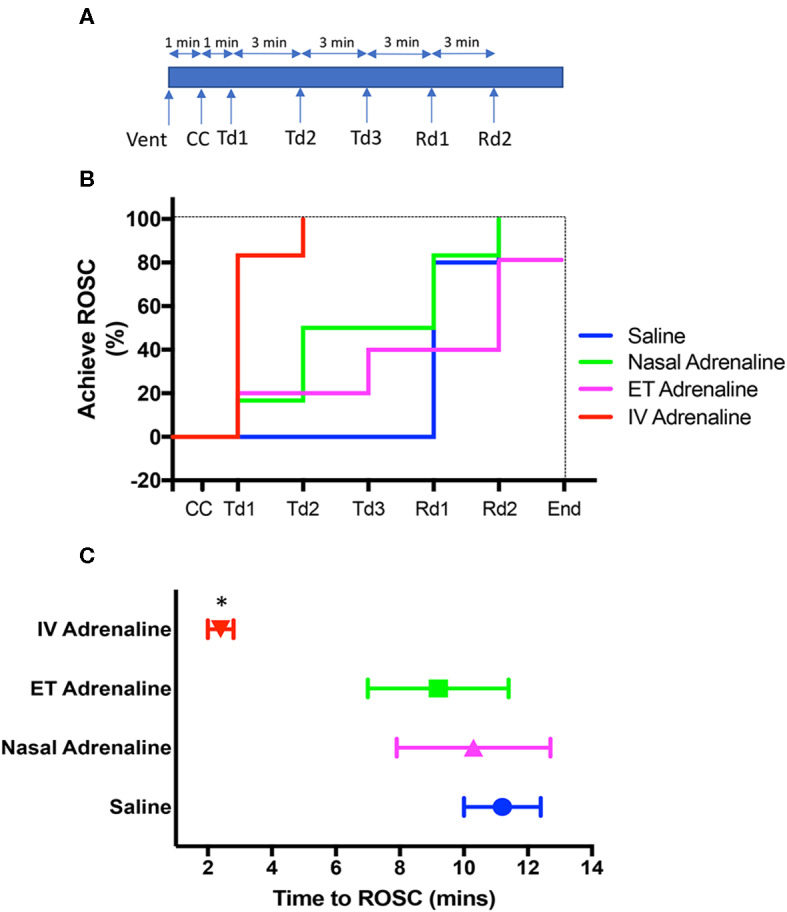
**(A)** Experimental time line from the cessation of carotid blood flow, showing the timing of ventilation onset (Vent) followed by chest compression (CC) onset as well as the timing of each treatment dose (Td) via allocated route or intravenous rescue dose (Rd) of adrenaline. Following the onset of Vent, CCs commenced 1 min later and was followed by the first adrenaline Td 1 min after that. Subsequent Tds and Rds were given at 3-min intervals as required until the return of spontaneous circulation (ROSC). **(B)** Survival curves showing the percentage of lambs in each treatment group achieving ROSC with each Td via allocated route intravenous (IV) saline, blue; Nasal Adrenaline, green; Endotracheal Adrenaline (ET; magenta); IV-Adrenaline (red) or IV Rd and overall survival at end of experiment. **(C)** The time taken to achieve ROSC in IV Adrenaline (red), Nasal Adrenaline (green), ET-adrenaline (magenta), and IV saline (blue) lambs. *Indicates that IV Adrenaline lambs significantly different to IV-saline, ET-adrenaline, and Nasal-adrenaline lambs. Data shown as mean±SEM.

**Figure 2:**
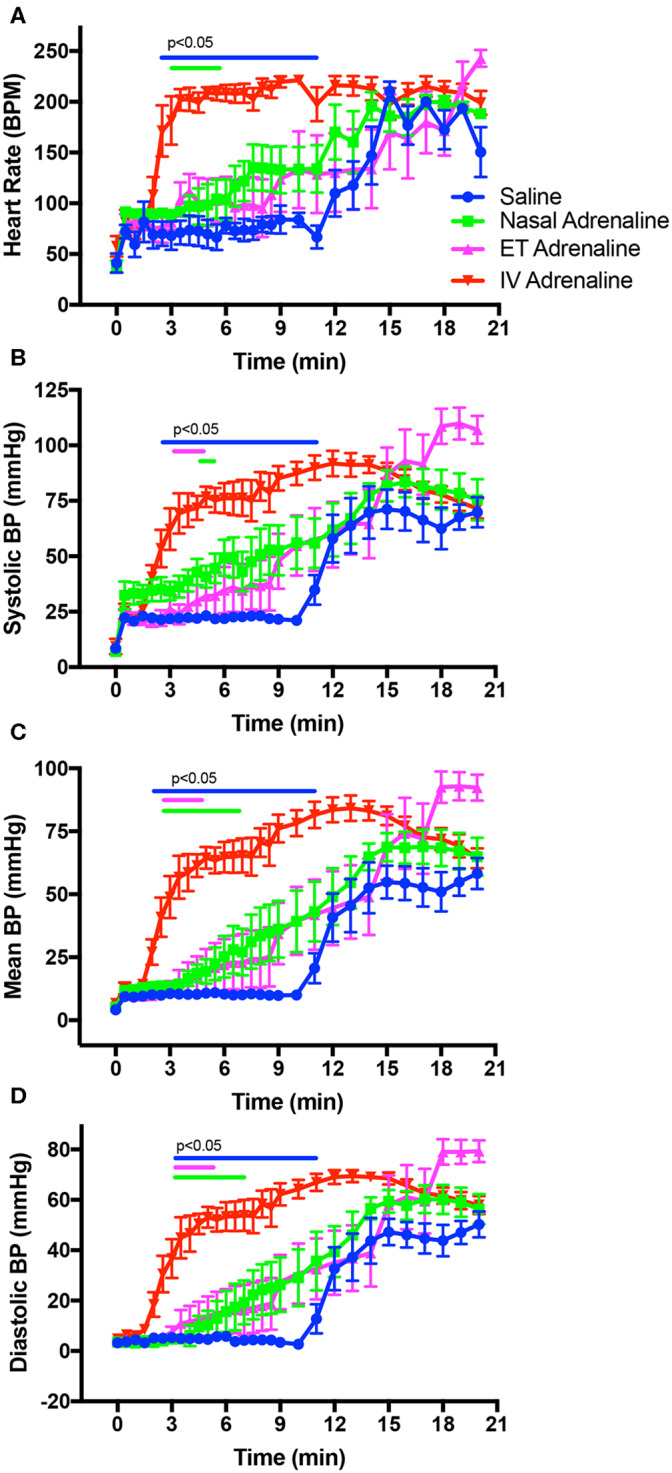
Changes in heart rate **(A)**, systolic blood pressure (BP; **B**), mean arterial BP **(C)**, and diastolic BP **(D)** in intravenous (IV)-saline (blue), Nasal-adrenaline (green), Endotracheal-adrenaline (ET; magenta), and IV-adrenaline (red) lambs over the first 21 mins following the onset of chest compressions (at time zero). Timing ranges indicated by the blue, green, and magenta bars designate values in IV-adrenaline lambs that were significantly greater (*p* < 0.05) than in IV-saline, Nasal-adrenaline, and ET-adrenaline lambs, respectively. Values in the IV-saline, Nasal-adrenaline, and ET-adrenaline lambs were not different from each other.

Lambs randomized to the ET-Adr or Nasal-Adr groups also received 5.0 mL of saline via the jugular vein—the same volume as lambs in the IV-Adr and IV-saline groups received. The first treatment dose (Td) was administered at 60 s after initiation of CCs (Figure 1A). Uncoordinated CCs were discontinued after ROSC. If ROSC was not achieved, the assigned treatment was repeated at 3 and 6 min following the first administration (Figure 2A). If there was no ROSC at 9 min, a rescue dose (Rd) of IV adrenaline (0.5 mL of 0.1 mg/mL) was administered, which was repeated at 12 min if ROSC still had not occurred. Experiments were stopped if no ROSC was achieved at 15 min, and the lambs were euthanized. Following ROSC, lambs were sedated with alfaxalone (4 mg/kg/h; Alfaxan, Jurox, NSW, Australia) and a maintenance infusion of glucose solution (50 mg/mL) was given at 6 mL mL/h. Animals were euthanized with an overdose of sodium pentobarbitone—ewes after delivery and lambs that achieved ROSC 30 min following the first dose of adrenaline.

### Monitoring

Carotid and pulmonary arterial blood flow (CBF/PBF; PowerLab; ADInstruments, Castle Hill, NSW, Australia), carotid arterial BP (DTX Plus Transducer; Becton Dickinson, Singapore), heart rate (HR) with electrocardiogram, rectal temperature, and SpO_2_ were recorded continuously prior to delivery and until the end of the experiment. Arterial blood samples were collected before cord clamping, at end of asphyxia, and at 3, 6, 9, 12, 15, and 20 min following the first dose of adrenaline and measured for pH, partial pressure of oxygen and carbon dioxide, oxygen saturation, base excess (BE), and lactate (ABL30, Radiometer, Copenhagen, Denmark). Plasma samples for adrenaline measurements were collected before asphyxia, at end of asphyxia, and at 3 and 15 min following the first dose of adrenaline (Adrenaline concentrations were determined using an enzyme immunoassay [Adrenaline Research ELISA, LDN, Germany], catalogue #BA E-5100). It must be noted that we also tested other available adrenaline ELISA kits and found many of them to be unreliable, which could be a species-related problem (sheep vs. human). Before making our measurements with the Adrenaline Research ELISA, LDN, Germany kit, we included a validation test, which involved spiking plasma samples from newborn lambs with known concentrations of adrenaline to ensure that we could precisely measure what we added. Plasma samples for cardiac troponin 1 measurements were collected before cord clamping, at end of asphyxia, and at 15 and 30 min. Troponin 1 concentrations were determined using the TROPI method, an automated colorimetric method carried out on a Beckman Coulter SYNCHRON LX20PRO System(s) with reagents and calibrators supplied by Beckman/Coulter (Sydney, Australia), catalogue #33340.

### Outcome Measures

Physiological parameters were measured from the start of CC and until ROSC, which was defined as a heart rate >100 bpm and mean carotid BP > 30 mmHg. These included mean, systolic and diastolic BP, mean PBF, mean, systolic and diastolic CBF, HR, and blood gas status. In addition, we recorded the number of adrenaline doses given to achieve ROSC, the need for IV adrenaline Rds, and failure to achieve ROSC after maximum adrenaline administration.

### Analytical Methods

All results are shown as means ± standard error of the mean (SEM). The Shapiro–Wilk test was used to assess frequency distribution, and data were normalized when required. A mixed model fitted by the restricted maximum likelihood (REML) method with a compound symmetry covariance matrix (GraphPad Prism 8) was used to analyze physiological parameters and plasma concentrations of adrenaline and troponin 1 data between treatment groups (IV-Adr, ET-Adr, nasal-Adr, and IV-saline) over time. Pairwise comparisons were made at specific time points and adjusted for multiple comparisons using Tukey's *post hoc* test. A linear regression for the rate of increase in select physiological parameters after ROSC was performed; see Figure 4. A one-way analysis of variance (ANOVA) was used to compare lamb demographics and select peak physiological measurements after ROSC, blood gas data, time taken to achieve ROSC, and total dose of adrenaline given to lambs to achieve ROSC between treatment groups. Statistical significance was defined as a *p*-value of < 0.05.

## Results

Twenty-four lambs were initially instrumented, but two lambs were later excluded for technical reasons. Baseline characteristics and physiological parameters before umbilical cord occlusion, at end of asphyxia, and 15 min after starting resuscitation for lambs in each group are shown in Table 1. There were no significant differences in HR, BP, and arterial blood gases measured between the groups before cord occlusion. There were no significant differences in HR and BP, but there were marginal differences in the measured PaO_2_, SaO_2_, and pH observed between groups at the end of asphyxia (Table 1). As the measured values were well outside the normal physiological range and were at or beyond the calibration range for the blood gas analyzer, these differences are not regarded as biologically important. After 15 min following ROSC, all blood gas values were similar (data not shown).

**Table 1:** Baseline characteristics and physiological parameters before cord occlusion, at end of asphyxia and 15 min after resuscitation and ventilation protocol started.

	**IV-Adrenaline (*n* = 6)**	**ET-Adrenaline (*n* = 5)**	**Nasal-Adrenaline (*n* = 6)**	**IV-Saline (*n* = 5)**	***p*-value**
Weight (kg)	4.2 ± 0.3	4.0 ± 0.3	3.8 ± 0.4	3.9 ± 0.3	NS
Female: Male	3:3	2:3	3:3	3:2	NS
**BEFORE CORD OCCLUSION (FETAL VALUES)**					
Heart rate (BPM)	138 ± 8	135 ± 4	157 ± 4	155 ± 10	NS
Mean arterial BP (mmHg)	47.7 ± 2.6	51.4 ± 3.9	53.5 ± 2.5	52.2 ± 2.0	NS
pH	7.24 ± 0.03	7.26 ± 0.01	7.20 ± 0.02	7.27 ± 0.03	NS
BE (mmol/L)	−4.9 ± 2.0	−2.9 ± 1.6	−4.4 ± 0.9	−2.7 ± 1.7	NS
PaO_2_ (mmHg)	17.2 ± 1.8	17.8 ± 2.1	18.8 ± 2.1	16.9 ± 1.3	NS
SaO_2_ (%)	39.4 ± 5.0	40.4 ± 5.9	39.4 ± 6.4	47.2 ± 6.9	NS
PaCO_2_ (mmHg)	54.0 ± 3.4	56.1 ± 2.0	63.2 ± 2.4	54.7 ± 2.8	NS
Lactate (mmol/L)	5.5 ± 1.5	4.1 ± 0.5	4.5 ± 0.4	5.8 ± 0.8	NS
**AT END OF ASPHYXIA**					
Heart rate (BPM)	66 ± 11	36 ± 6	41 ± 8	41 ± 11	NS
Mean arterial BP (mmHg)	4.9 ± 1.2	6.7 ± 1.1	6.6 ± 1.2	5.1 ± 1.2	NS
pH	6.90 ± 0.01^a^	6.88 ± 0.02^ab^	6.83 ± 0.02^b^	6.93 ± 0.01^a^	<0.05
BE (mmol/L)	−16.9 ± 0.9	−15.9 ± 1.2	−17.8 ± 0.7	−15.5 ± 0.7	NS
PaO_2_ (mmHg)	3.2 ± 0.6^a^	2.9 ± 0.2^a^	5.4 ± 0.5^b^	2.7 ± 0.7^a^	<0.05
SaO_2_ (%)	3.5 ± 0.5^a^	3.9 ± 0.7^ab^	6.4 ± 0.7^b^	7.9 ± 3.5^ab^	<0.05
PaCO_2_ (mmHg)	111 ± 6.4	118 ± 3.3	124 ± 5.2	108 ± 3.4	NS
Lactate (mmol/L)	11.7 ± 0.8	11.2 ± 0.4	11.9 ± 0.2	12.9 ± 1.1	NS
**15 MIN AFTER STARTING RESUSCITATION PROTOCOL**					
Heart rate (BPM)	197 ± 15	170 ± 43	186 ± 18	210 ± 11	NS
Mean Arterial BP (mmHg)	81.2 ± 3.7	68.8 ± 15.4	68.7 ± 6.1	54.8 ± 7.8	NS
pH	7.20 ± 0.04	7.00 ± 0.07	7.05 ± 0.11	7.11 ± 0.05	NS
BE (mmol/L)	−14.3 ± 1.1^a^	−21.2 ± 1.5^b^	−17.9 ± 0.7^ab^	−17.7 ± 1.2^ab^	<0.05
PaO_2_ (mmHg)	210 ± 88	210 ± 80	166 ± 40	306 ± 87	NS
SaO_2_ (%)	98.3 ± 0.5	87.4 ± 9.6	90.1 ± 7.6	95.1 ± 3.3	NS
PaCO_2_ (mmHg)	33.0 ± 5.3	49.1 ± 10.2	54.7 ± 15.9	38.2 ± 4.9	NS
Lactate (mmol/L)	10.3 ± 1.2	11.5 ± 1.1	11.9 ± 0.8	12.6 ± 0.7	NS

*Values are presented as mean ± SEM. p-values >0.05 are considered not significant (NS). Values that do not share a common letter (a,b) are significantly different from each other p < 0.05. BPM beats per minute, BP blood pressure, BE base excess, PaO_2_ arterial partial pressure of oxygen, PaCO_2_ arterial partial pressure of carbon dioxide, SaO_2_ arterial oxygen saturation, ET endotracheal, IV intravenous*.

### ROSC

In the IV-Adr group, 5/6 lambs achieved ROSC after the first Td; the remaining lamb required two Tds before achieving ROSC (Figure 1B). In the ET-Adr group, 1/5 lambs required one Td, 1 lamb required three Tds, 2 lambs required 2 Rds, and 1 did not achieve ROSC even after the second Rd. In the Nasal-Adr group, 1/6 lambs required one Td, 2 required two Tds, whereas 3 lambs required either one (2 lambs) or two (one lamb) Rds of adrenaline before they achieved ROSC. In the IV-saline group, no lambs achieved ROSC until after administration of adrenaline Rds; 4/5 lambs required one Rd, and 1 lamb required two Rds (Figure 1B). Time from initiation of CCs until ROSC was significantly shorter in the IV-Adr group (2.4 ± 0.4 mins) compared with the ET-Adr (10.3 ± 2.4 mins), Nasal-Adr (9.2 ± 2.2 mins), and IV-saline (11.2 ± 1.2 mins) groups (Figure 1C). There were no significant difference in time to ROSC between the ET-Adr, Nasal-Adr, and IV-saline groups.

### Cardiovascular Parameters During Chest Compressions

**Figure 3:**
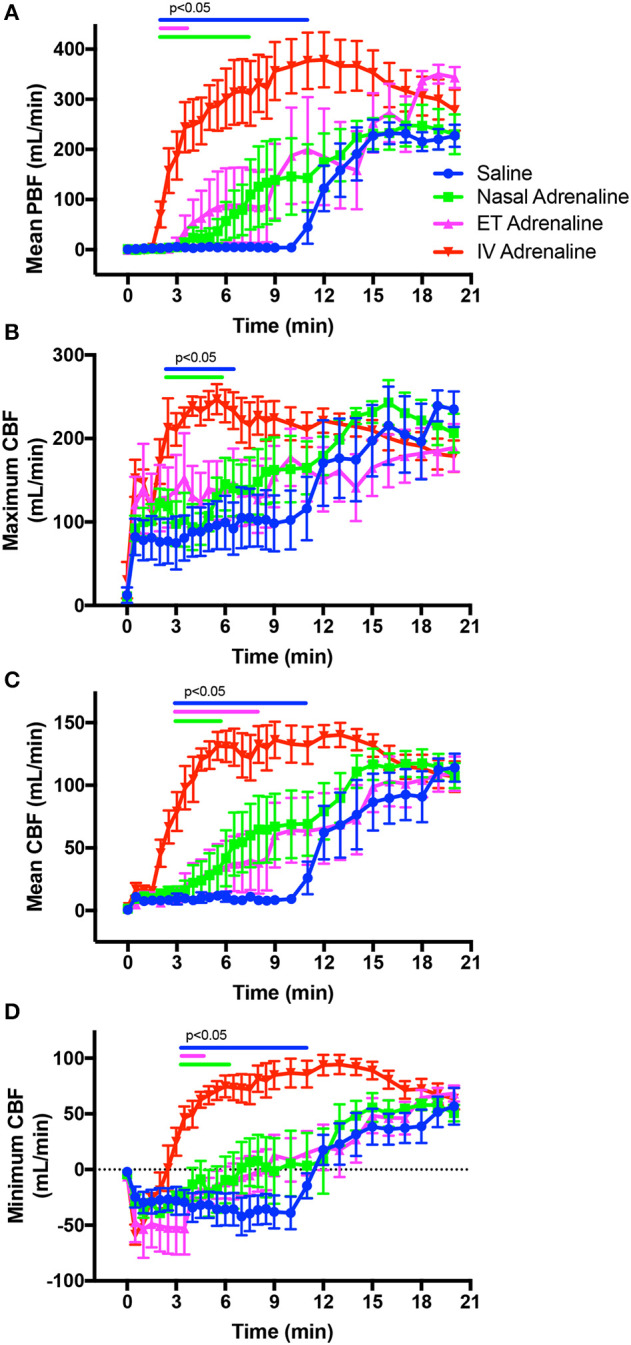
Changes in mean pulmonary blood flow (PBF; **A**), maximum (systolic) carotid arterial blood flow (CBF; **B**), mean CBF **(C)**, and minimum (diastolic) CBF **(D)** in intravenous (IV)-saline (blue), Nasal-adrenaline (green), Endotracheal-adrenaline (ET; magenta), and IV-adrenaline (red) lambs over the first 20 mins following the onset of chest compressions (at time zero). Data ranges indicated by the blue, green, and magenta bars designate values in IV-adrenaline lambs that are significantly (*p* < 0.05) greater than in IV-saline, Nasal-adrenaline, and ET-adrenaline lambs, respectively. Values in the IV-saline, Nasal-adrenaline, and ET-adrenaline lambs were not different from each other.

Before ROSC, the rate of CCs (measured as HR) was similar between groups, varying between 70 and 90 compressions/min over the first 30 s (Figure 2A). While CCs were able to achieve systolic BP above 20 mmHg in most animals after one min of CCs, the diastolic BP remained low (Table 2, Figures 2B–D). CCs were able to generate significant forward CBF toward the brain (Table 2, Figure 3B), but during relaxation all lambs had retrograde CBF away from the brain, as indicated by negative values (Table 2, Figure 3D). As a result, at one min after the start of CC and before ROSC, the mean CBFs were low and PBFs also remained low (Table 2, Figures 3A,C).

**Table 2:** Cardiovascular parameters 1 min after the onset of chest compression before adrenaline is given and during the recovery phase following ROSC.

	**IV-Adrenaline (*n* = 6)**	**ET-Adrenaline (*n* = 5)**	**Nasal-Adrenaline (*n* = 6)**	**IV-Saline (*n* = 5)**	***p*-value**
**BLOOD PRESSURE AND BLOOD FLOW 1 MIN AFTER ONSET OF CHEST COMPRESSIONS**					
Systolic BP (mmHg)	22.7 ± 2.5	21.8 ± 2.8	33.2 ± 5.0	20.2 ± 2.0	NS
Mean BP (mmHg)	11.7 ± 2.0	9.8 ± 1.4	12.1 ± 1.1	9.2 ± 0.9	NS
Diastolic BP (mmHg)	6.0 ± 2.0	4.1 ± 0.9	3.2 ± 1.1	4.4 ± 0.7	NS
CBF during relaxation (negative = retrograde flow)	−47.4 ± 3.9	−53.3 ± 25.9	−37.2 ± 6.3	−29.6 ± 9.7	NS
Mean CBF (mL/min)	15.7 ± 4.3	10.4 ± 4.4	11.4 ± 3.0	7.5 ± 2.5	NS
Mean PBF (mL/min)	−1.1 ± 3.5	2.4 ± 2.2	0.9 ± 1.1	2.6 ± 0.7	NS
**INCREASE IN BLOOD PRESSURE, BLOOD FLOW, AND HEART RATE FOLLOWING ROSC**					
Rate of increase in mean arterial BP (mmHg per min)	11.0 ± 1.4^a^	4.1 ± 0.6^b^	5.6 ± 0.3^b^	9.0 ± 1.7^a^	<0.05
Rate of increase in CBF (mL/min per min)	27.7 ± 2.5^a^	7.5 ± 1.0^c^	6.5 ± 0.7^c^	14.2 ± 2.4^b^	<0.05
Rate of increase in PBF (mL/min per min)	52.8 ± 5.9^a^	20.6 ± 3.3^c^	17.3 ± 0.9^c^	34.7 ± 3.5^b^	<0.05
Rate of increase in HR (BPM)	64.2 ± 10.0^a^	7.1 ± 1.3^c^	5.7 ± 1.5^c^	32.5 ± 5.1^b^	<0.05
**REBOUND HYPERTENSION AFTER ROSC**					
Peak systolic BP (mmHg)	96.0 ± 5.0	91.5 ± 17.6	93.5 ± 8.0	86.5 ± 6.5	NS
Peak mean BP (mmHg)	87.7 ± 4.5	83.1 ± 16.1	77.9 ± 6.8	67.3 ± 4.9	NS
Systolic BP at 30 mins (mmHg)	63.4 ± 6.7^a^	90.9 ± 14.3^b^	65.2 ± 6.6^ab^	76.8 ± 8.0^ab^	<0.05
Mean BP at 30 mins (mmHg)	53.3 ± 6.3	77.2 ± 1.8	57.3 ± 6.7	65.5 ± 7.7	NS

*Values are presented as mean ± SEM. p-values >0.05 are considered not significant (NS). Values that do not share a common letter (a,b,c) are significantly different from each other p < 0.05. BPM beats per minute, BP Blood pressure, CBF carotid artery blood flow, PBF pulmonary blood flow, HR heart rate, IV intravenous, ET endotracheal, ROSC return of spontaneous circulation*.

### Cardiovascular Parameters Following ROSC

**Figure 4:**
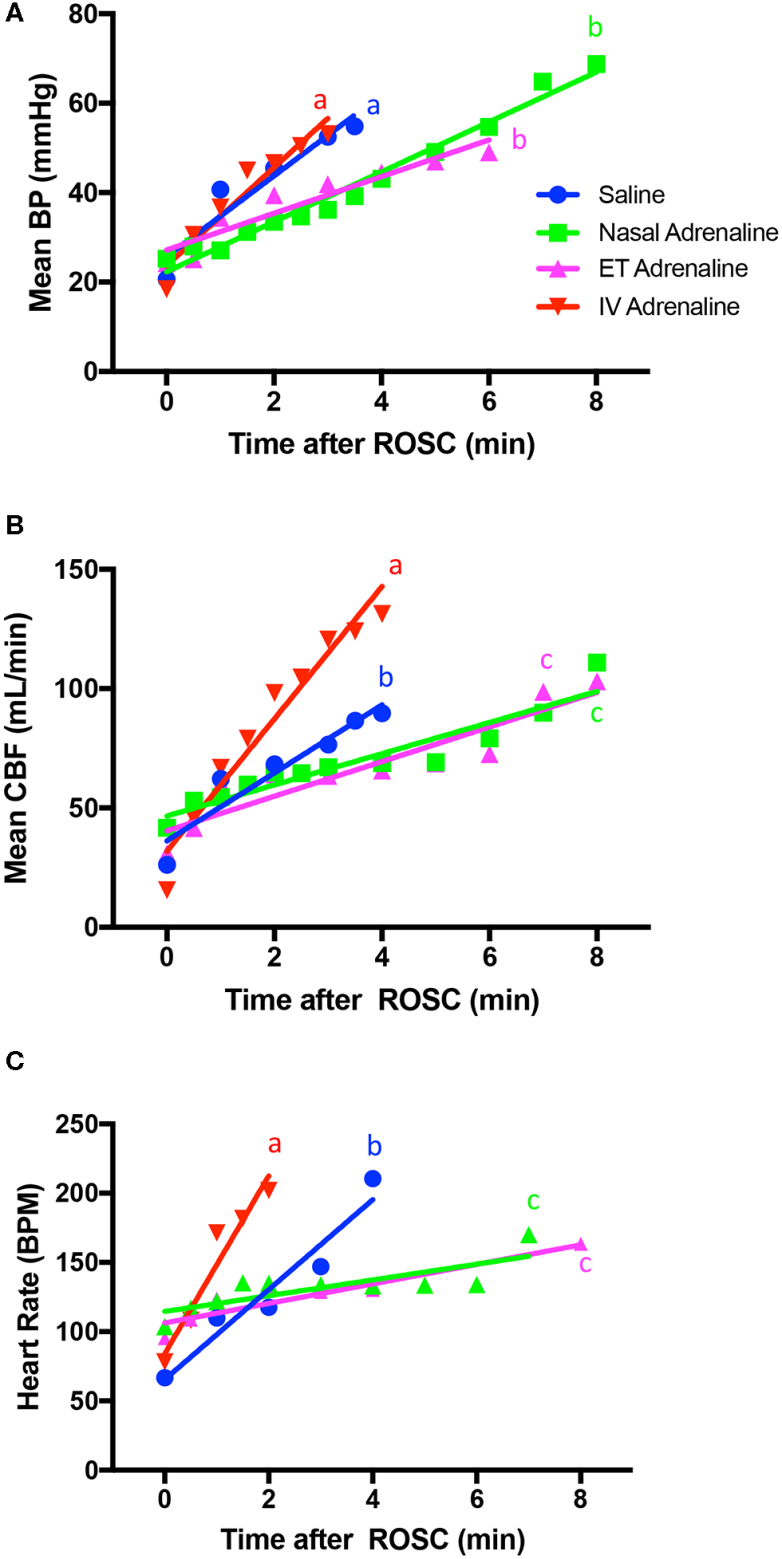
Rate of increase in mean arterial blood pressure (BP; **A**), mean carotid arterial blood flow (CBF; **B**), and heart rate **(C)** after the return of spontaneous circulation (ROSC) in intravenous (IV)-saline (blue), Nasal-adrenaline (green), Endotracheal-adrenaline (ET; magenta), and IV-adrenaline (red) lambs. Regression lines that do not share a common letter are statistically significantly different (*p* < 0.05).

Following ROSC, the rate of increase in mean arterial BP was significantly greater in IV-Adr lambs compared to ET-Adr and Nasal-Adr lambs, but was similar to IV-saline lambs who also only received IV adrenaline Rds (Table 2, Figure 4A). The rate of increase in CBF and PBF following ROSC was significantly greater in IV-Adr lambs compared to ET-Adr and Nasal-Adr lambs (Table 2, Figures 4B,C). However, the rates of CBF and PBF increase in IV-saline lambs were less than in IV-Adr lambs but greater than in both ET-Adr- and Nasal-Adr-treated lambs (Table 2, Figure 4B). Similarly, the rate of increase in HR following ROSC (Table 2, Figure 4C) was significantly greater in IV-Adr lambs compared to ET-Adr, Nasal-Adr, and IV-saline lambs, with the increase in IV saline lambs (receiving IV adrenaline Rds) being significantly greater than in both ET-Adr and Nasal-Adr lambs (Table 2, Figure 4D).

The rebound hypertension and tachycardia that followed ROSC appeared greater in IV-Adr lambs than in Nasal-Adr- and IV-saline-treated lambs (Table 2, Figures 2A,B). However, the highest-peak mean and systolic BPs were reached in ET-Adr-treated lambs compared to all other groups and remained high until the end of the experiment. However, these higher BPs were not associated with higher CBFs in ET-Adr lambs, as CBFs were similar between all groups at 20 mins after the start of CCs (Figure 3C). While PBFs tended to be elevated in ET-Adr-treated lambs, at 20 mins after starting CCs, this difference was not significant (Figure 3D).

**Figure 5:**
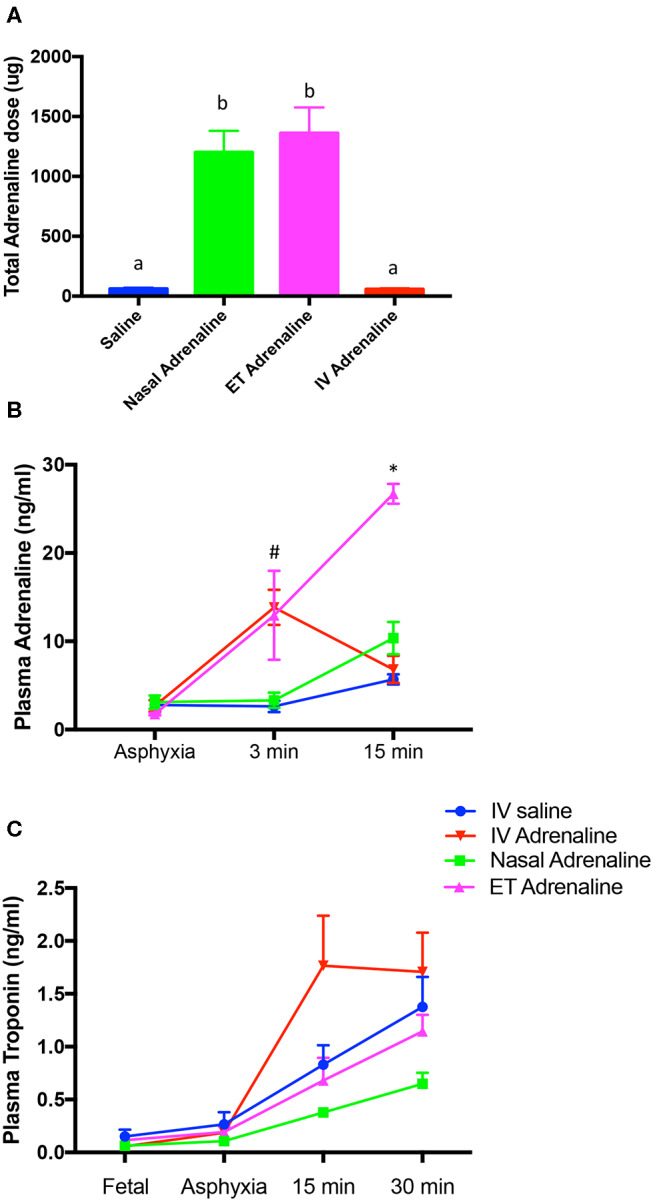
Total dose of adrenaline administered to achieve return of spontaneous circulation (ROSC; **A**) and plasma Adrenaline **(B)** and Troponin 1 **(C)** concentrations measured in plasma from IV-saline (blue), Nasal-adrenaline (green), Endotracheal-adrenaline (ET; magenta), and IV-adrenaline (red) lambs. In **(A)**, values that do not share a common letter are significantly (*p* < 0.05) different from each other. In **(B)**, ^#^Indicates that adrenaline concentrations are significantly (*p* < 0.05) greater in IV-adrenaline compared to IV-saline and Nasal-adrenaline but not ET-adrenaline lambs; *Indicates that adrenaline concentrations in ET-adrenaline lambs are significantly greater than IV-saline, IV-adrenaline and Nasal-adrenaline lambs. In **(C)**, plasma troponin 1 levels were significantly (*p* < 0.05) higher in IV-adrenaline lambs overall, but no individual time points were different.

The total dose of adrenaline administered was significantly greater in Nasal-Adr- and ET-Adr-treated lambs compared with IV-saline- and IV-Adr-treated lambs (Figure 5A), due to the 10-fold dose given by these routes. At 3 mins after initiating CCs, circulating adrenaline concentrations were significantly higher in IV-Adr- and ET-Adr-administered lambs than in Nasal-Adr- and saline-treated lambs (Figure 5B). However, 15 mins after initiating CCs, plasma adrenaline concentrations were increased further in ET-Adr-treated lambs but were similar in IV-Adr-, Nasal-Adr-, and saline-treated lambs (Figure 5B). Circulating troponin 1 levels were significantly elevated following asphyxia, but were increased to a much greater extent at 15 mins in the IV-Adr lambs (Figure 5C). While IV-saline- and ET-Adr-treated lambs had similar troponin 1 concentrations at each time point, troponin 1 concentrations were significantly lower in Nasal-Adr-treated lambs.

## Discussion

Acute administration of adrenaline by IV or ET routes during newborn resuscitation may be delayed due to the technical challenges of establishing IV access or performing ET intubation. In an attempt to develop a simple, alternate route for rapid administration of adrenaline, we evaluated whether adrenaline can be administered via the nasal route during resuscitation of severely asphyxiated near-term lambs. We found that in asystolic lambs receiving CCs, ROSC was only achieved after adrenaline administration, which is consistent with our previous observations (8). The IV route of administration of adrenaline was more effective than nasal and ET routes. However, the cardiovascular responses to nasal and ET administered adrenaline were similar; 50% of the Nasal-Adr-treated lambs achieved ROSC with 1 or 2 Tds, whereas 40% of the ET-Adr-treated lambs achieved ROSC after 3 Tds. Furthermore, of the 3 ET-Adr-treated lambs that required Rds of IV adrenaline, all required 2 Rds and ROSC was not restored in one lamb. In contrast, all Nasal-Adr-treated lambs requiring Rds achieved ROSC after 1 or 2 Rds.

There is a lack of studies investigating adrenaline administration during cardiopulmonary resuscitation of infants or animals in the min following delivery (5). Adrenaline administration in severely asphyxiated 1-day-old (i.e., post-transitional) newborn pigs did not improve circulatory recovery in animals already receiving ventilation and CC (17, 18). The authors suggested that high endogenous catecholamine levels or severe acidosis explained why early adrenaline administration did not further improve outcome (17, 18). In contrast, in our study all lambs receiving CC, ventilation, and IV adrenaline achieved ROSC. However, none of the lambs receiving CC and ventilation alone (saline group) achieved ROSC until Rds of IV adrenaline were given. Similarly, all but one lamb receiving either ET or nasal adrenaline achieved ROSC following adrenaline administration, given as either Td or Rd. Of the 11 lambs requiring IV Rds of adrenaline, 10 achieved ROSC, with 6 lambs requiring one dose and 4 lambs requiring two doses. We found that adrenaline was essential in achieving ROSC in all newborn lambs after severe asphyxia and was effective even when administered IV 11 mins following the start of CC. Similar results were obtained by Vali et al. where 39/44 severely asphyxiated lambs achieved ROSC after IV adrenaline (19).

Not only did the onset of ROSC occur sooner but also the rates of increase in BP, HR, CBF, and PBF were all significantly greater in IV-Adr lambs than in both ET-Adr and Nasal-Adr lambs (Figure 5). It is interesting that the rate of increase in BP pressure was similar in both saline and IV-Adr lambs, but as all saline lambs were given IV rescue doses of adrenaline, this is not surprising. Nevertheless, the recovery of BP in saline lambs occurred after 12 mins of CCs, whereas in IV-Adr lambs the BP increase commenced after 2.5 mins of CCs. However, while the rates of increase in CBF, PBF, and HR following ROSC were significantly greater in saline lambs than in ET-Adr and Nasal-Adr lambs, they were significantly less than in IV-Adr lambs. The lower rate of increase in CBF and PBF in saline lambs, compared to IV-Adr lambs, despite similar BPs, is likely due to the lower rate of HR increase. However, this raises an interesting dilemma because we have previously shown that the increase in CBF following ROSC is largely pressure passive (8) and so we would have expected the rate of increase in CBF to be similar in saline and IV-Adr lambs. However, the finding that the increase in CBF was less, despite similar increases in pressure, suggests that cerebral vascular resistance was higher during ROSC in saline lambs than in IV-Adr lambs. The reason for this is unclear, but may result from a protracted period of very low BP in the IV-saline group, compared to the IV-Adr group (~2.5 vs. ~12 mins), which led to spontaneous vasoconstriction of arterial vessels in the absence of an internal distending pressure. As such, it is possible that, in response to a period of severe asphyxia that results in myocardial failure, the cerebral vascular response is bimodal. Initially, the vessels are maximally dilated to increase flow and maintain cerebral oxygen delivery, but following a prolonged period of very low or absent arterial pressure, the vessels spontaneously contract resulting in an increase in resistance following ROSC.

While it is likely that an earlier ROSC is beneficial, one may question whether the very rapid rate of restoration in cardiovascular function observed in IV-Adr lambs is potentially harmful. Indeed, we have previously suggested that a rapid increase in cardiac function will rapidly elevate BPs, which exposes the maximally vasodilated cerebral microvascular bed to high pressures and flows leading to micro hemorrhages (15). In addition, it is interesting that troponin levels were also highest in IV-Adr lambs, whereas these lambs presumably had the shortest exposure to poor coronary perfusion and anoxic conditions. This result raises the question as to whether the higher workload and contraction rate required to more rapidly restore cardiovascular function in the IV-Adr group may also increase myocardial injury. Indeed, a higher workload and contraction rate require a higher oxygen demand, but myocardial tissue oxygen stores (myoglobin) were unlikely to be replenished and contractions temporarily reduce myocardial blood flow and oxygen delivery. Similar marked increases in troponin levels have been reported after the use of adrenaline in severely asphyxiated human infants (20). It is also of interest that Nasal-Adr lambs had the lowest troponin levels and clearly lower than the ET-Adr lambs possibly due to the sustained high adrenaline levels in ET-Adr lambs driving a high HR and cardiac output.

Data on appropriate adrenaline doses in neonates are sparse. In asphyxiated 1-day-old piglets, a standard dose was found to be as equally effective as a high dose of adrenaline, when outcomes were assessed at 4 h, but mortality was increased in the high-dose group at 24 h (21, 22). Similarly, other animal studies have suggested that high doses (100 μg/kg) of adrenaline may be harmful, reducing stroke volume and cardiac output in hypoxic and bradycardic neonatal lambs (23). In our study, the total dose of adrenaline given was significantly greater in ET-Adr and Nasal-Adr lambs largely because of the need to give multiple treatment doses and the fact that a 10-fold higher dose was given when administering adrenaline via the ET or nasal route. We found that circulating adrenaline concentrations were significantly higher in ET-Adr lambs compared to Nasal-Adr lambs at 3 mins after starting CCs and were not statistically different from IV-Adr lambs. This was largely due to high adrenaline levels (26.1 ng/mL) in one ET-Adr lamb which also happened to be the lamb that required only one Td. This finding is consistent with the concept that increased circulating adrenaline levels are required to achieve ROSC and is consistent with the findings of Vali et al. showing that a rapid ROSC is associated with a rapid increase in plasma adrenaline concentrations (19). However, it also suggests that the circulating adrenaline concentrations required to achieve ROSC may have a threshold which must be exceeded before ROSC will be triggered. Nevertheless, there is cause for concern that adrenaline levels continued to increase in ET-Adr lambs after ROSC and likely remained elevated. This result was reflected by and likely explains the markedly higher mean, diastolic, and systolic BPs at 20 mins after initiating CCs that persisted until 30 mins (data not shown). This finding indicates that adrenaline uptake across the respiratory epithelium is effective, but that the uptake is somewhat delayed, resulting in a cumulative dosing effect, due to the failure to achieve ROSC within an appropriate time frame.

In a retrospective study from a large US tertiary center, ET adrenaline at dosages of 30–50 μg/kg appeared less efficacious than IV adrenaline (10 μg/kg) during neonatal resuscitation (12). In contrast, a small retrospective study from a tertiary center in New Zealand found that ET adrenaline was associated with a favorable response in the majority (28/39) of infants receiving one or two doses by this route (24). In the asphyxiated newborn infant prior to successful transition, delayed clearance of airway liquid may dilute the adrenaline or impair/delay the time taken for adrenaline to reach the alveoli from where it can be taken up into the circulation (4). On the other hand, we found that Nasal-Adr administration at a dose of ~100 μg/kg resulted in significantly lower plasma adrenaline concentrations than IV-Adr and ET-Adr lambs at 3 min. Nevertheless, 50% of severely asphyxiated lambs achieved ROSC following 2 doses of Nasal-Adr, and as such if concentrations were measured between 6 and 9 mins after CCs, it is possible that adrenaline levels would have been elevated. Nasal adrenaline administration has been evaluated in other acute post-neonatal animal models (25–28), but to our knowledge never in a transitional newborn asphyxia model. In our study, nasal adrenaline administration was simple and fast, and the cardiovascular response in Nasal-Adr lambs was similar to ET-Adr. We noted that ROSC was achieved at least as well using the nasal route compared with ET-Adr (50% with 2 Tds vs. 40% with 3 Tds). However, both routes were less effective than IV administration. Optimizing intranasal drug therapy requires techniques including minimizing drug volume while maximizing drug concentration, adequate dosing, use of both nostrils to double the absorptive mucosal surface, and use of atomized particles to enhance drug absorption (29). Concentrated medications in a small volume (0.2–0.3 mL per nostril), like those used in our study, are suggested to be optimal (29). In this experiment, the first dose of adrenaline was given at the same time in all animals, regardless of mode of administration. However, in a clinical setting it is likely that nasal adrenaline could be given several minutes earlier, as nasal adrenaline can be administered long before an ET-tube or UVC is placed. In particular, in a setting where skilled emergency teams are not present at birth, this alternative has a potential benefit.

In this study, lambs were resuscitated using an initial sustained inflation before ventilation at a rate of 60/min and uncoordinated CC 90/min was started. This differs somewhat from current recommended guidelines for resuscitation of newborn infants (1, 7). However, as this was an experiment designed to compare the effectiveness of adrenaline administration via different routes, we opted to use an established lamb asphyxia model shown to be effective in previous experiments in asphyctic lambs (15).

The current study has strengths and limitations. Our model mimics the transitional pathophysiology of a newborn in the delivery room, in which resuscitation following asphyxial arrest occurs in the context of physiological changes including lung aeration, initially high then falling pulmonary vascular resistance, and vascular shunts including patent ductus arteriosus. There was a significant difference in response between lambs given IV adrenaline and the control group receiving IV saline. The intermediate response after ET and nasal adrenaline suggests that both routes may have a role in neonatal resuscitation, in particular before IV access is established. We appreciate that there are differences in reported adrenaline concentrations after adrenaline administration in animal “cardiac arrest” models, and in some studies these concentrations are not reported (17, 22). The adrenaline concentrations in our study were lower than those reported by Vali et al. (19). However, in line with Vali et al., we observed the same striking clinical response with ROSC in animals given IV adrenaline (19). Differences in animal models, dosing schedules, and assays may partly explain the observed discrepancies in measured adrenaline concentrations.

## Conclusion

The IV route of adrenaline administration is more effective than either nasal or ET routes. Nasal administration had similar effects as ET administration and is an easier route for early adrenaline administration. The use of nasal adrenaline administration during neonatal resuscitation merits further investigation.

## Data Availability Statement

The raw data supporting the conclusion of this article will be made available by the authors, without undue reservation, to any qualified researcher.

## Ethics Statement

The animal study was reviewed and approved by The Monash University, Animal Ethics Committee (MMCA).

## Author Contributions

NS designed the study, reviewed the literature, collected and analyzed the data, and wrote a first draft. CK conceived and designed the study, reviewed the literature, analyzed the data, wrote a first draft, and submitted the manuscript. EM and GS participated in the study design and data collection and edited the manuscript. GP and VZ participated in the study design, data collection, and analysis and edited the manuscript. PD participated in the study design and data interpretation and edited the manuscript. SH planned the study, participated in the study design, collected and analyzed the data, carried out data interpretation, and edited the manuscript. KC wrote the ethics applications, planned the study, coordinated the study, participated in the study design, collected and analyzed the data, and edited the manuscript. All authors have read and approved the final manuscript as submitted and agreed to be accountable for all aspects of the work.

## Conflict of Interest

The authors declare that the research was conducted in the absence of any commercial or financial relationships that could be construed as a potential conflict of interest.

## Abbreviations

BP: Blood pressure
CBF: Carotid blood flow
CC: Chest compressions
ET: Endotracheal
HR: Heart rate
IV: Intravenous
PBF: Pulmonary blood flow
Rd: Rescue dose adrenaline IV
ROSC: Return of spontaneous circulation
Td: Treatment dose adrenaline - by the assigned route.
